# Clozapine-induced Agranulocytosis: A Case Presentation and Literature Review

**DOI:** 10.1192/j.eurpsy.2025.2078

**Published:** 2025-08-26

**Authors:** P. A. Hernández Liebo, M. Polo Gay, J. Romay González, C. Sevilla Díez, I. Ibarra Muñoz, M. de la Fuente Gómez, A. Herrán Gómez, J. Vázquez Bourgon

**Affiliations:** 1 Psychiatry, University Hospital Marqués de Valdecilla; 2 Hospital Universitario Marqués de Valdecilla, Santander, Spain

## Abstract

**Introduction:**

Clozapine has the strongest evidence for efficacy for schizophrenia that has proved refractory to adequate trials of standard antipsychotic medication. Its use is limited to these cases due to the uncommon but severe adverse effect agranulocytosis or severe neutropenia, defined as a neutrophil count under 500/microL. It is seen in 0,4 % of clozapine patients and it usually occurs in the first three months. Risk is managed with close blood count (BC) monitoring protocol.

**Objectives:**

This work aims to improve the understanding and management of this condition.

**Methods:**

With this purpose, we present a clinical report and rewiew its management in literature.

**Results:**

We present the case of a 60-year-old man with the diagnosis of resistant schizophrenia who is hospitalized in the acute Psychiatry Unit due to decompensation, where clozapine is initiated with gradual dose augmentation and weekly BC. After improvement of psychotic symptoms, the patient is transferred to a subacute care facility. Two months later, BC revealed mild neutropenia (1000/microL; defined as 1000-1500/microL) becoming severe (100/microL) on the next test one week later. Clozapine is then interrupted and replaced with olanzapine. Neutrophils descend to zero within one day and three days later, with all granulocytes in low levels, the patient presents fever and diarrhea, being finally hospitalized in Internal Medicine. Empirical intravenous antibiotic therapy is prescribed as well as filgastrim, a granulocite-colony stimulating factor (G-CSF). Antibiotic is adjusted after *Enterococo Faecalis* is isolated in blood cultives. Despite eleven days without clozapine and eight days with G-CSF, agranulocytosis persisted. Taking in consideration the severity of the case and the non existence of acute psychotic symptoms, olanzapine is interrupted and benzodiacepine medication is increased, with BC normalization within three days and remission of digestive symptoms and fever. G-CSF is interrupted and the patient is re-transferred to the subacute unit, with initiation of aripiprazol in the following days.

Upon the appereance of mild neutropenia, closer blood monitorization is recommended (three times a week); with interruption of clozapine and daily monitoring in case of moderate or severe neutropenia. In relation to when to reintroduce antipsychotic treatment, clinical guidelines are cautious, emphasizing the need of close hematological monitoring and careful evaluation of risks and benefits. However, most recommend waiting for complete neutrophil recovery, taking into account the severity of the psychotic symptoms. Olanzapine, with similar mechanism of action but much lower risk, is usually the election.

**Image 1:**

**Figure:**
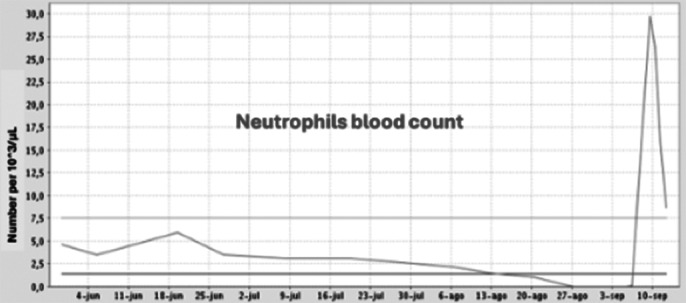

**Image 2:**

**Figure:**
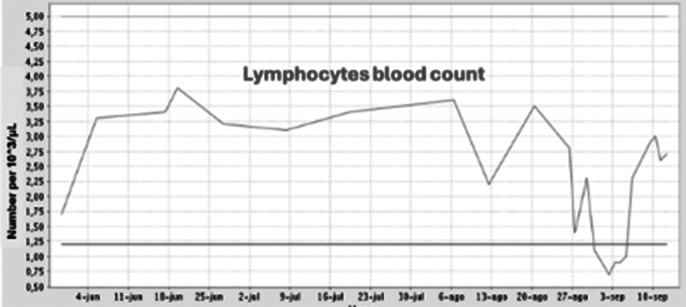

**Image 3:**

**Figure:**
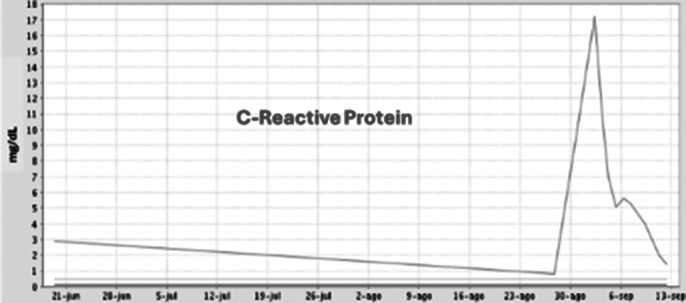

**Conclusions:**

Although clozapine-induced agranulocytosis is rare, its complexity and severity requires a fine review of its management, considering that this probably has an impact on the patient’s clinical evolution.

**Disclosure of Interest:**

None Declared

